# Supplementary material for: Splice-Junction-Based Mapping of Alternative Isoforms in the Human Proteome
Source: Cell Rep. Author manuscript; Available in PMC 2020 Jan 15. (PMC6961840; doi:10.1016/j.celrep.2019.11.026)
Supplement: 2 [file NIHMS1546469-supplement-2.zip › DF1/PXD006675/RightVentricle/RightVentricle_6_TNS1_TPLSALGLKPHNPADILLHPTGEEDEGK.pdf]

A

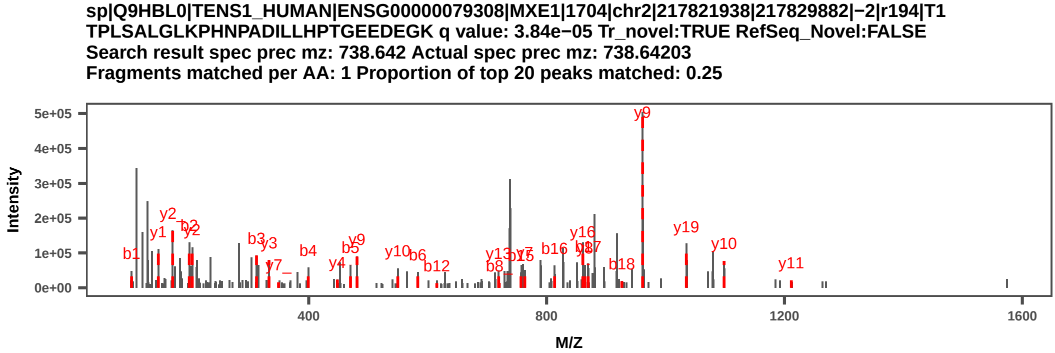
sp|Q9HBL0|TENS1_HUMAN|ENSG00000079308|MXE1|1704|chr2|217821938|217829882|−2|r194|T1
TPLSALGLKPHNPADILLHPTGEEDEGK q value: 3.84e−05 Tr_novel:TRUE RefSeq_Novel:FALSE
Search result spec prec mz: 738.642 Actual spec prec mz: 738.64203
Fragments matched per AA: 1 Proportion of top 20 peaks matched: 0.25
Intensity
M/Z

B

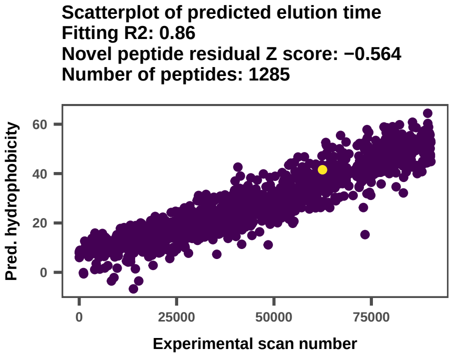
Scatterplot of predicted elution time
Fitting R2: 0.86
Novel peptide residual Z score: −0.564
Number of peptides: 1285
Pred. hydrophobicity
Experimental scan number

C

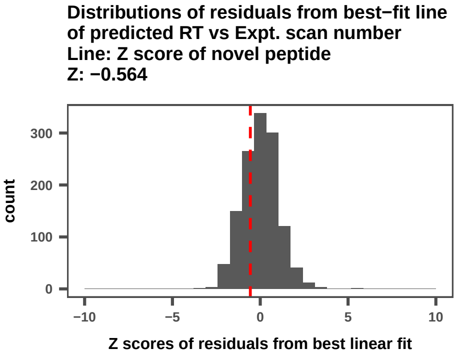
Distributions of residuals from best−fit line
of predicted RT vs Expt. scan number
Line: Z score of novel peptide
Z: −0.564
count
Z scores of residuals from best linear fit
